# Biosorption of Triphenyl Methane Dyes (Malachite Green and Crystal Violet) from Aqueous Media by Alfa (*Stipa tenacissima* L.) Leaf Powder

**DOI:** 10.3390/molecules28083313

**Published:** 2023-04-08

**Authors:** Lamia Ouettar, El-Khamssa Guechi, Oualid Hamdaoui, Nadia Fertikh, Fethi Saoudi, Abudulaziz Alghyamah

**Affiliations:** 1 Laboratory of Organic Synthesis Modeling and Optimization of Chemicals, Department of Process Engineering, Faculty of Technology, Badji Mokhtar–Annaba University, P.O. Box 12, Annaba 23000, Algeria; 2 Department of Process Engineering, Faculty of Technology, Badji Mokhtar–Annaba University, P.O. Box 12, Annaba 23000, Algeria; 3 Chemical Engineering Department, College of Engineering, King Saud University, P.O. Box 800, Riyadh 11421, Saudi Arabia

**Keywords:** biosorption, triphenylmethane dyes, Alfa leaf powder, kinetics, isotherm, modeling

## Abstract

This study includes the characterization and exploitation of an abundant agricultural waste in Algeria, Alfa (*Stipa tenacissima* L.) leaf powder (ALP) as a biosorbent for the removal of hazardous triphenylmethane dyes, malachite green (basic green 4) and crystal violet (basic violet 3), from aqueous media under various operating conditions in batch mode. The effect of experimental parameters such as initial dye concentration (10–40 mg/L), contact time (0–300 min), biosorbent dose (2.5–5.5 g/L), initial pH (2–8), temperature (298–328 K), and ionic strength on dye sorption was investigated. The results of both dyes show that the increase in initial concentration, contact time, temperature, and initial pH of solution leads to an increase in biosorbed quantity, unlike the effect of ionic strength. The biosorption kinetics for triphenylmethane dyes on ALP was analyzed by pseudo-first-order, pseudo-second-order, Elovich, and intraparticle diffusion models proposed by the Weber–Morris equation. Equilibrium sorption data were analyzed by six isotherms, namely the Langmuir, Freundlich, Harkins–Jura, Flory–Huggins, Elovich, and Kiselev isotherms. The thermodynamic parameters were evaluated for both dyes. The thermodynamic results suggest that both dyes’ biosorption is a typical physical process, spontaneous and endothermic in nature.

## 1. Introduction

According to the United World Water Development Report, 2,000,000 tons of wastes are discharged to the receptor water bodies every day, including industrial wastes, dyes, chemicals, etc. [1]. The increased usage of synthetic dyes in industries has occasioned in production of effluents that contain highly toxic and carcinogenic compounds, high chemical oxygen demand, and total organic carbon (TOC) [2]. The presence of dyes in water resources not only has an aesthetic impact, but also reduces the amount of sunlight that reaches water bodies, which inhibits photosynthetic activity and ultimately destroys aquatic ecosystems [3]. Furthermore, the colors in water harm people’s health by seriously harming their kidneys and central nervous systems [4,5]. It is extremely noticeable and unpleasant to have even very small concentrations of colors in water (less than 1 ppm for certain colors) [6]. Dyes are carcinogenic and possess a serious hazard to aquatic living organisms [7], and the metabolites of many dyes have toxic teratogenic and mutagenic effects on humans and aquatic life, due to the presence of aromatics, metals, chlorides, etc. [6,8]. Cationic dyes are basic dyes, having bright colors, and are extensively used in industries [9]. In particular, basic dyes, such as triphenylmethane, are considered to be one of the more problematic classes of dyes [4]. Crystal violet (CV) and malachite green (MG) are important water-soluble basic dyes belonging to triphenylmethane family, which are widely used for coloring purposes and which were selected in this study as typical representative pollutants. Both dyes have the potential to permanently harm both human and animal eyes, in addition to causing digestive system and skin discomfort [10]. In addition, it is well established that MG and CV cause liver tumors and are extremely cytotoxic and carcinogenic to mammalian cells. Therefore, it is necessary to reduce dyes concentration in the wastewater before it is released into the environment. There are several techniques for removing dyes from aqueous solutions; however, from an economic standpoint, these methods are mostly expensive in underdeveloped nations [11]. In order to accomplish this, new techniques to remove synthetic colors from wastewater are continuously being developed. Biosorption technology is getting more popular as a desirable and promising technology [12,13,14].

Retention of pollutants such as dyes, metal ions, and others occurs during the biosorption process on solid biologically derived materials known as biosorbents [15,16]. Consequently, adsorbents or sorbents made from various types of biomass, mostly industrial and agricultural waste, are known as biosorbents. van der Waals attraction, electrostatic attraction, complexation, ion exchange, covalent binding, adsorption, and microprecipitation are only a few of the many processes that lead to biosorption [14,16,17]. From an environmental point of view, the study of biosorption, which uses natural materials or industrial and agricultural wastes, is very important since it may be thought of as an alternate technique for removing dangerous contaminants [4,9,11,14,16].

Alfa plant (*Stipa tenacissima* L.) or esparto grass (Alfa is the Arab name for esparto) [18] is a perennial plant with rapid growth that does well in arid climates of North Africa and Southeast Spain [19]. It is a member of the *poaceae* family. Its long, fiber-rich leaves can be as long as 1 m. The leaves are firm, smooth, shiny, ribbon-like, thin, and have a hairy sheath at the base. Depending on the location and environmental circumstances, the leaves of an Alfa plant reach maturity between the fourth and twelfth month after blossoming. Alfa grass has two growth seasons (fall and spring) and two latent seasons in its biological cycle (winter and summer). The Alfa grass tufts are fully vegetative in the fall. As a result, the majority of the leaves are mature, while the younger ones begin to grow and lengthen. The majority of leaves are young in winter, though, and the cold weather stunts their growth. The Alfa plant thrives in a wide range of bioclimatic conditions and is resistant to large variations in temperature. The uses of this plant are very diverse. Indeed, because it is a rich source of cellulose, it is used for its fibers in paper manufacturing and as a low-cost composite material for reinforcement [18]. The dimensional stability of sheets manufactured from the Alfa plant with variations in moisture content is another crucial feature. Additionally, this facility is employed in the production of rope, carpets, and handcrafted espadrilles and baskets. Due to its extensive root system, which holds and safeguards the earth, this plant also acts as a priceless natural barrier that prevents the desert from spreading. The quantity of extractives in hot water (Table 1 [20]) is relatively high when compared to wood and other annual plants, and the amount of ash in the Alfa plant is around of 3%, which is relatively high when compared to that of wood. The composition of this mineral component was analyzed, evidencing that the main atoms contained in Alfa plant ash are silicone (more than 50%), magnesium, aluminum, calcium, iron, and sodium (around of 15%) [20].

The aim of the present study is to examine the applicability of Alfa (*Stipa tenacissima* L.) leaf powder as a biosorbent in the removal of the hazardous basic dyes, malachite green and crystal violet, from aqueous media in a batch process. Firstly, the effects of different parameters including initial dye concentration, contact time, biosorbent dose, initial pH, temperature, and ionic strength on the removal of dyes were investigated. Subsequently, the biosorption kinetics for both dyes at different concentrations was determined by the pseudo-first-order, pseudo second-order, and Elovich models. Additionally, the biosorption mechanism was analyzed using the Weber–Morris model. Six isotherm models—Langmuir, Freundlich, Harkins–Jura, Flory–Huggins, and Kiselev—were used to examine equilibrium sorption data. Furthermore, thermodynamic parameters were evaluated for both dyes. Lastly, the biosorption parameters obtained with the current biosorbent will be compared to those described in the literature.

## 2. Results

### 2.1. Characterization of ALP

**Figure 1:**
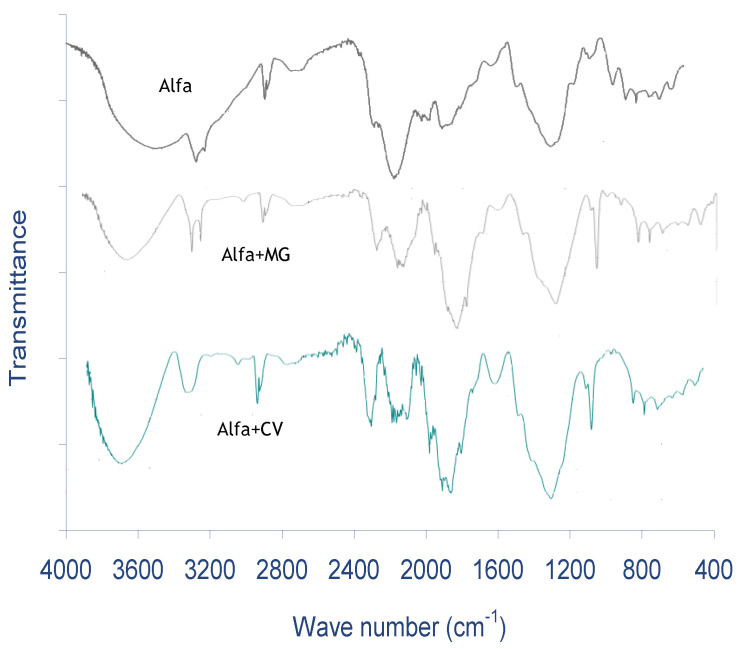
FTIR spectrum of Alfa leaf powder (ALP).

The obtained FTIR spectra revealed that there were various functional groups identified on the raw ALP surface (Figure 1). The strongest peak detected at 3480 cm^−1^, found in the ALP spectrum, could be assigned to O–H stretching of phenol groups of cellulose and lignin. Other peaks detected on the ALP were –CH and –CH_2_ stretching of aliphatic compounds and N-H stretching or C=O stretching, respectively, recorded at 2900, 2850, and 2360 cm^−1^. Additionally, other functional groups observed on the ALP biosorbent at 1636, 1420, and 1263 cm^−1^ were attributed, respectively, to C=C stretching of phenol groups, CH_2_ deformation, and Si–C stretching or C–O stretching of phenol groups. On the other hand, the peaks located at 1110, 1029, 661, and 518 cm^−1^ are assigned to C–N stretching, stretching of ether groups of cellulose, twist C-O-H group, and C-S stretching, respectively. There were novel peaks detected after MG and CV biosorption, whose intensities were weaker than those obtained before the biosorption. It is clear that the Alfa biosorbent displays several surface functional groups reflecting the diverse character of the Alfa surface and can be contributed positively to its dye removal efficiency.

The Boehm titration method is a chemical technique used to recognize basic and acid surface groups on the ALP. The result of this method (Table 2) attested the nature of the ALP surface, which enhanced the biosorption of the basic dyes because these results show that the total acid groups were higher than the total basic groups. Similar results have been reported in the literature [11,12,14,21,22,23]. From acid groups, the carbonylic and quinonic and after phenolic groups were the dominant acidic oxygenated groups. Consequently, these results explicit the nature of the ALP surface.

The point of zero charge (pH_pzc_) of a biosorbent is the pH at which the surface of the biomaterial has an electrical neutrality. The sorbent surface has a net negative charge at pH > pH_pzc_, though at pH < pH_pzc_, the surface has a net positive charge. The pH_pzc_ of ALP was calculated using the solid addition method [23,24,25] and was determined to be 6.3 (Table 3). Similar results (pH_pzc_ 6.8) were observed for Alfa stems [26] and for meranti sawdust (pH_pzc_ 6.23) [25]; however, for the extracted Alfa fibers, a pH_pzc_ value of 7.7 was obtained [27].

**Figure 2:**
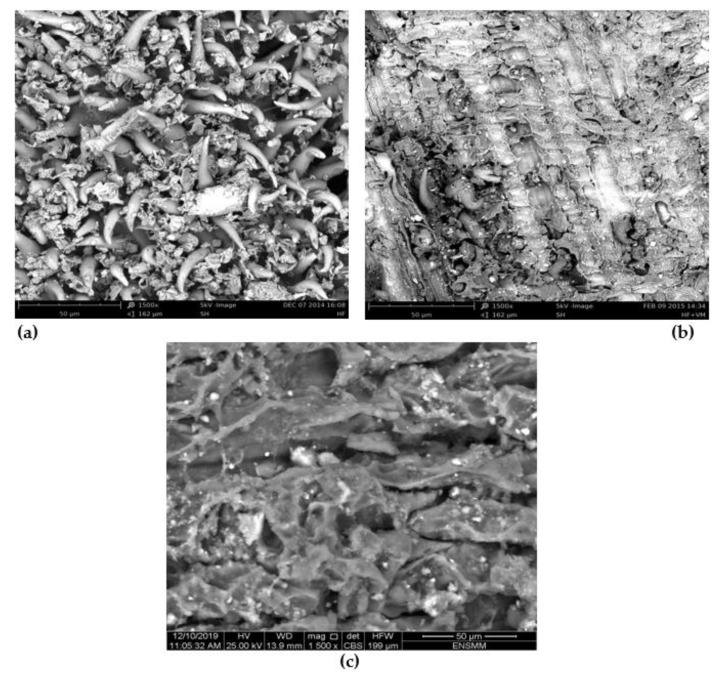
SEM micrograph (×1500) of ALP before (**a**) and after (**b**) MG and (**c**) CV biosorption.

The SEM image of the ALP biosorbent particles is shown in Figure 2a (1500× magnification). This figure shows irregular texture and a heterogeneous surface with the presence of spines like claws on ALP particles’ surface, which indicates a certain degree of roughness and also porous structure. It can be observed that the morphology of the ALP surface undergoes a change after biosorption of MG (Figure 2b, 1500× magnification) or CV (Figure 2c, 1500× magnification) with the disappeared rough texture after interaction with both dyes (see Figure 2b,c), indicating a smooth surface texture of ALP by comparing with the surface morphology before biosorption of dyes (Figure 2a).

The X-ray diffraction technique is an effective method for determining if a substance is crystalline. In crystalline materials, well-defined peaks are visible; however, in non-crystalline or amorphous systems, a hollow peak appears instead of a well-defined peak [28]. The XRD analysis (figure not shown) of the ALP pattern displays no apparent diffraction peaks, which indicates a characteristic shape of ALP that is amorphous in nature. In the literature, similar results were reported for other biosorbents [14,29].

**Table 3:** Principal characteristics of Alfa leaf powder (ALP).

Component	Value
Apparent specific gravity (g/cm^3^)	0.29
Absolute specific gravity(g/cm^3^)	1.25
Porosity (%)	76.8
Point of zero charge (pH_PZC_)	6.3
Porosity (%)	76.8
Specific surface area—BET (m^2^/g) [30]	3.28
Fiber average diameter (µm) [31]	90−120

### 2.2. Influence of Operating Conditions

#### 2.2.1. Effect of Initial Dye Concentration and Contact Time

**Figure 3:**
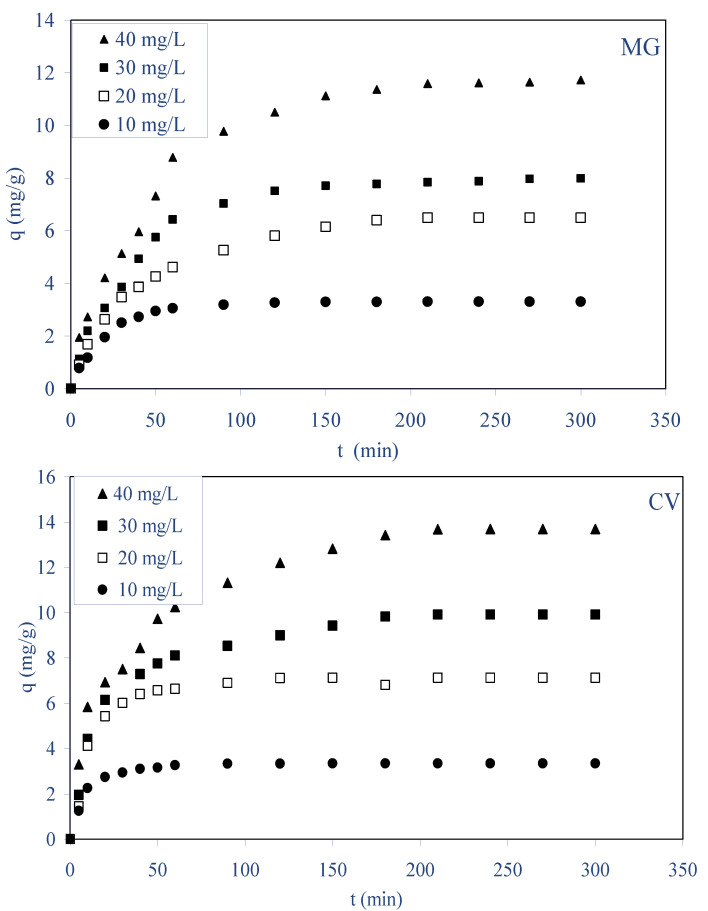
Effect of initial concentration and contact time on the biosorption of MG and CV dyes on ALP (C_0_: 10–40 mg/L; ALP dosage: 2.5 g/L; stirring speed: 400 rpm; pH (MG): 4, pH (CV): 6.5, T: 298 K).

In batch biosorption processes, the initial dye concentration in the solution plays a key role as a driving force to overcome the mass transfer resistance between the solution and solid phases. Figure 3 illustrates the biosorption for both dyes at different initial concentrations (10–40 mg L^−1^) for a fixed biosorbent dose of 2.5 g/L as a function of contact time (0–300 min) for a stirring speed of 400 rpm at 298 K and natural initial pHs for MG and CV. The plots of Figure 3 can be divided into three distinct regions: (i) rapid biosorption during the first 5–50 min, (ii) gradual equilibrium until the equilibrium state for each concentration, and (iii) the equilibrium state. For both dyes, the result indicates that an increase in initial dye concentration leads to an increase in the biosorption by ALP. This observation could be explained by the theory that in the process of dyes biosorption, initially the MG and CV dye molecules have to first encounter the boundary layer effect and then diffuse from the boundary layer film onto the biosorbent surface, and then finally they have to diffuse into the porous structure of the biosorbent [32]. At equilibrium, the sorbed amount increased for MG from 3.23 to 11.70 mg g^−1^ and for CV from 3.55 to 13.75 mg g^−1^ as the concentration increased from 10 to 40 mg L^−1^, respectively. The resistance to the uptake for both dyes from the solution decreases with the increase in dyes concentration, and this is due to increasing concentration gradient, which acts as an increasing driving force to overcome all mass transfer resistances between the aqueous solution and solid phase [33]. The necessary time to reach equilibrium increased from 40 to 150 min and from 30 to 180 min, respectively, for MG and CV while the initial dye’s concentration increased from 10 to 40 mg L^−1^, respectively. This is because biosorption sites sorbed accessible dye molecules more quickly at low concentrations, whereas dyes had to diffuse to the interior sites of the biosorbent at high concentrations. Similar observations were reported for the sorption a number of dyes by diverse material biosorbents [12,22,23,33,34,35].

#### 2.2.2. Effect of Biosorbent Dosage

**Figure 4:**
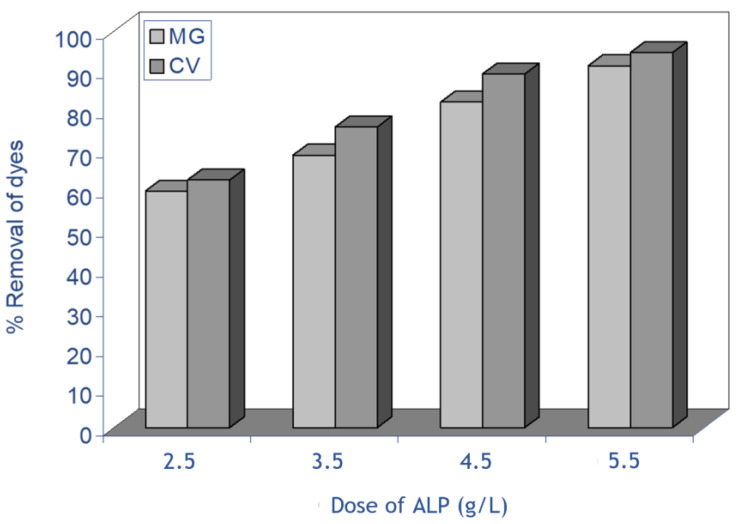
Effect of biosorbent dosage on the removal of both dyes on ALP (conditions: C_0_: 40 mg/L; biosorbent dosage: 2.5−5.5 g/L; stirring speed: 400 rpm; pH (MG): 4, pH (CV): 6.5, T: 298 K).

The experiments of effect of biosorbent dosage on the removal by biosorption of MG and CV on ALP were conducted for different mass in the range of 2.5–5.5 g/L, using an initial dye concentration of 50 mg L^−1^. Figure 4 shows the effect of biosorbent dose on the biosorption of both dyes, and it was observed that an increase in the ALP dose resulted in increase in the removal percentage due to the increase in the number of biosorption sites, and this is due to greater availability of the exchangeable sites or surface area at a higher concentration of the biosorbent [14]. The percentage removal of MG biosorption at equilibrium increases from 59.7% to 91.3% for MG and from 62.5% to 94.63% for CV with an increase in biosorbent doses from 2.5 to 5.5 g/L, respectively. A similar observation was previously reported by many researchers for different dyes using low-cost adsorbents [25,35,36,37].

#### 2.2.3. Effect of Initial pH

**Figure 5:**
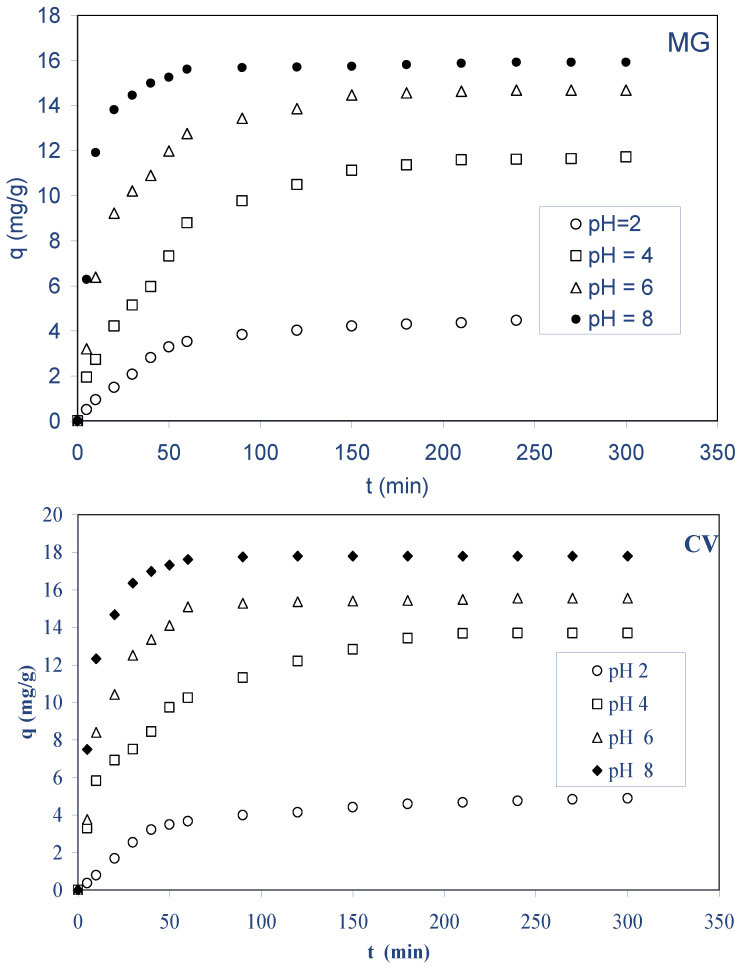
Effect of initial pH on the biosorption of MG and CVon ALP (conditions: C_0_: 40 mg/L; biosorbent dosage: 2.5 g/L; stirring speed: 400 rpm; pH: 2−8, T: 298 K).

The initial pH of the solution influences both the surface charge of the biosorbent and the degree of ionization of the components present. Since hydrogen and hydroxyl ions are significantly biosorbed, the pH of the solution influences the sorption of other ion dyes [38]. As a result, multiple experiments with variable pH values were performed, varying the starting pH of solutions between 2 and 8, using an initial dyes concentration of 40 mg L^−1^ and a biosobent mass of 2.5 g/L at 298 K. The biosorbed amount for both dyes on ALP at different pH values is plotted in Figure 5. The result from this figure reveals that the amount of MG increased from 4.57 to 15.98 mg g^−1^ and for CV from 4.92 to 17.81 mg g^−1^ with the increasing of solution pH from 2 to 8, respectively. It is often seen that the surface biosorbed anions favorably at lower pH due to the presence of H^+^ ions, whereas at higher pH due to the deposition of OH^−^ ions, the surface is active for the sorption of cations. Furthermore, on the basis of the point of zero charge, pH_pzc_, of the biosorbent, it was possible to explain the influence of the solution pH on the cationic dyes’ uptake. Knowing that the pH_pzc_ of ALP is 6.3, the surface charge of ALP is positive at pH < 6.3, neutral at pH equals 6.3, and negative at pH > 6.3 [39]. At lower pH values (2–4), the biosorbent becomes positively charged, and then the diminution in the biosorption is attributed to electrostatic repulsions existing between the surface of ALP and the cationic triphenylmethane dyes (MG and CV) cations in solution. However, at higher pH values, the biosorbent becomes negatively charged, and then the biosorption kinetics is attributed to electrostatic attraction. A similar behavior was observed in the literature [33,35].

#### 2.2.4. Effect of Temperature

**Figure 6:**
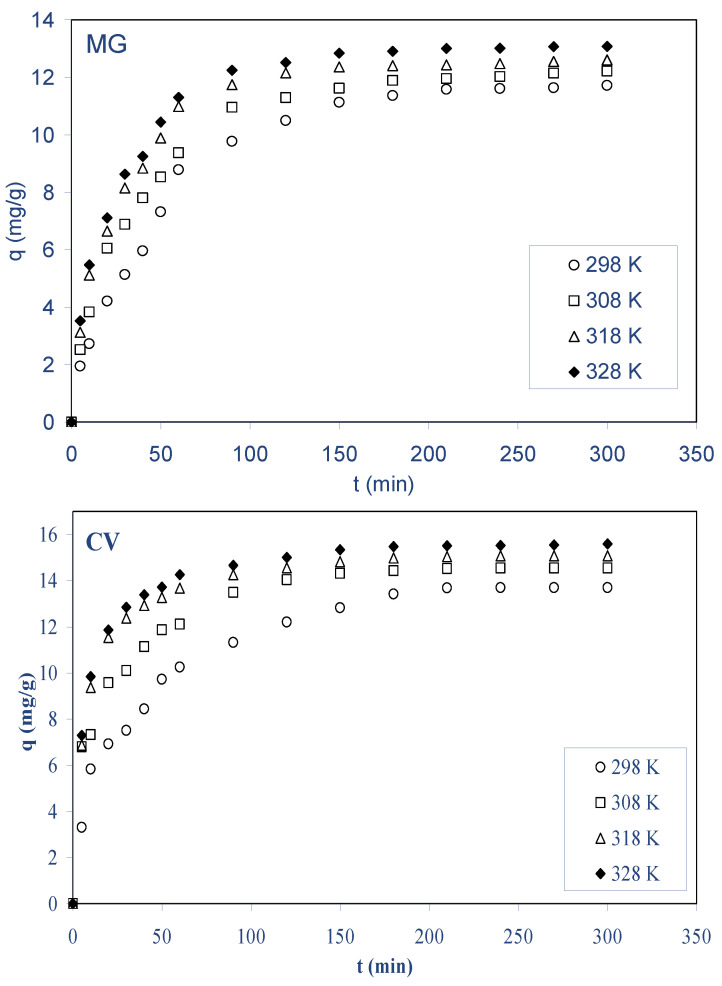
Effect of temperature on the biosorption of MG and CV on ALP (conditions: C_0_: 40 mg/L; biosorbent dosage: 2.5 g/L; stirring speed: 400 rpm; pH (MG): 4, pH (CV): 6.5, T: 298–328 K.

Temperature is a critical factor in the sorption process. Figure 6 depicts the biosorption of both dyes by ALP at various temperatures (298 to 328 K). For both basic dyes, it was shown that when the temperature rises from 298 to 328 K, the biosorption on ALP increases. The amount of dye biosorbed increases slightly from to 11.70 to 13.10 mg g^−1^ for MG and from 13.75 to 15.60 mg g^−1^ for CV, while the solution temperature increases from 298 to 328 K, respectively. This indicates that the overall biosorption process is endothermic in nature. As the temperature increased, the mobility of dyes increased, which caused an increase in the rate of diffusion of the dyes’ molecules across the boundary layer and in the internal pores of the ALP biosorbent particle, owing to the decrease in the viscosity of the solution. Several references have demonstrated that biosorption processes are enhanced by increasing the mobility of solute molecules [12,22,33].

The improved biosorption at higher temperature indicates endothermic biosorption of both dyes on ALP. It is recognized from Table 4 that ΔG° values were found to be negative, which indicates that the biosorption efficiency of MG and CV on ALP increases with increase in temperature. Gibbs free energy changes (ΔG°) are calculated using the following equations:

ΔG° = −R_g_T lnb_M_ (1)

where T (K) is the absolute temperature, R_g_ (8.314 J mol^−1^K^−1^) is the ideal gas constant, and b_M_ (L mol^−1^) is the Langmuir equilibrium constant. ΔG° (kJ mol^−1^) is the function of change in enthalpy, ΔH° (kJ mol^−1^), as well as change in standard entropy, ΔS° (J mol^−1^ K^−1^):

ΔG° = ΔH° − T ΔS°(2)

From the slope and the intercept of the plot of ΔG° versus T, ΔH° and ΔS° values were calculated. The calculated parameters by using the above equations were collected in Table 4. The negative values of ΔG°, from −1.99 to −2.61 kJ mol^−1^ for MG and from −15.24 to −18.25 kJ mol^−1^ for CV, demonstrate the feasibility of the process and the spontaneous nature of the biosorption with a high preference of CV on ALP. The positive values of ΔH° (5.52 kJ mol^−1^ for MG and 14.81 kJ mol^−1^ for CV) indicated the endothermic nature of the biosorption interaction. The lower and positive value of ΔS° (25.3 and 100.9 J mol^–1^ K^–1^), respectively, for MG and CV indicates that no remarkable change in entropy occurred during the biosorption of both dyes and especially for MG.

#### 2.2.5. Effect of Ionic Strength

**Figure 7:**
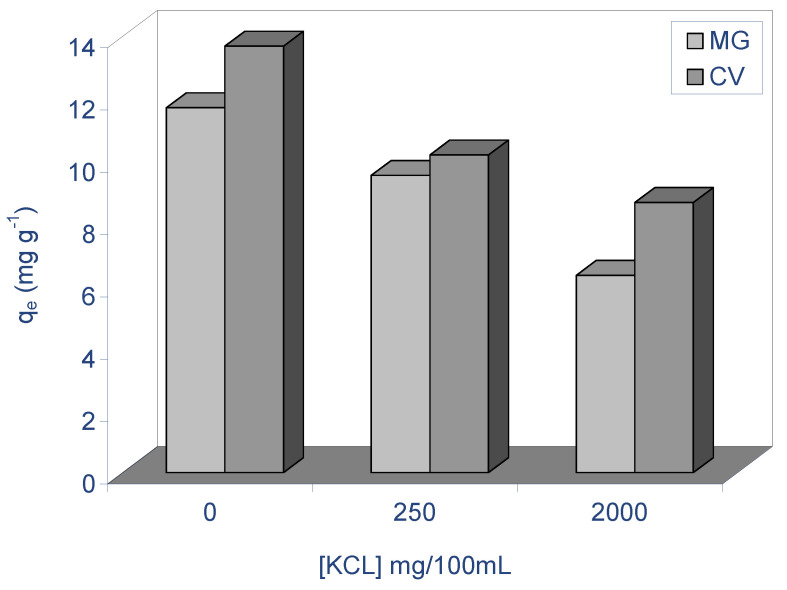
Effect of salt concentration (ionic strength) on the biosorption of MG and CV on ALP (conditions: C_0_: 40 mg/L; biosorbent dosage: 2.5 g/L; stirring speed: 400 rpm; pH (MG): 4, pH (CV): 6.5, T: 298 K).

Salts with elevated concentrations can frequently be found in colored wastewater, and the presence of salts or co-ions in solution can affect the biosorption of metal or dye ions. In order to study the biosorption aptitude of ALP towards cationic dyes in the presence of salts, KCl (potassium chloride) was chosen in this study with diverse ionic strengths. The influence of ionic strength on the biosorption of both dyes ions on ALP was examined using a constant initial concentration of 40 mg L^−1^, biosorbent mass of 2.5 g/L, temperature of 298 K, stirring speed of 400 rpm, different concentrations of KCl (0–2000 mg/100 mL), and natural pH for each dye. Figure 7 demonstrates that when the concentration of salt in the medium increased, the quantity of both dyes that were biosorbed decreased. While the concentration of KCl in the medium was increased from 0 to 2000 mg/100 mL, the biosorbed amount at equilibrium decreased from 11.70 to 6.33 mg g^−1^ for MG and from 13.75 to 8.67 mg g^−1^ for CV, respectively. This decrease in the biosorption capacity of ALP while the concentration of inorganic salt in the medium was increased is due to small size of salt ions compared to dyes’ molecules and compete with dyes for the biosorption sites [40,41]. Furthermore, this phenomenon can be explained by the fact that while the ionic strength was augmented, the electrical double layer surrounding the sorbent surface was compressed, which would lead to a diminution in the electrostatic potential in that order, resulting in a decrease of the Coulombic free energy and a reduction in MG and CV biosorption on ALP biosorbent [42,43]. Similar results have been reported for the biosorption of CV [41,42].

### 2.3. Modeling of Biosorption Kinetics

Diverse biosorption kinetic models have been used to describe the uptake of sorbate depending upon the time. In this study, the experimental data at different concentrations for the biosorption of MG and CV by ALP were fitted using the pseudo-first-order, pseudo-second-order, and Elovich models. The best appropriate model was selected based on the magnitude of the regression correlation coefficient (r) as a first condition, that is, the kinetic model giving a value of r adjacent to unity is considered to offer the best fit and the experimental amount biosorbed at equilibrium (q_e_) will be close up to the calculate the amount biosorbed as a second one.

#### 2.3.1. Pseudo-First-Order Model

The Lagergren [23] equation was used to investigate the suitability of the pseudo-first-order model to describe the biosorption of MG and CV by ALP. This equation can be written as:

ln (q_e_ − q_t_) = ln q_e_ − k_1_t (3)

where q_e_ (mg g^−1^) and q_t_ (mg g^−1^) are the amount of MG and CV biosorbed at equilibrium and at any time t, respectively, and k_1_ (min^−1^) is the rate constant. The plot of ln (q_e_−q_t_) versus t gives straight lines for the sorption kinetics at different initial dye concentrations (10, 20, 30, and 40 mg L^−1^) (figure not shown). The values of the pseudo-first-order rate constant k_1_ and the biosorbed amounts at equilibrium (q_e,cal_) were determined. The k_1_ values, the correlation coefficients, r, and the calculated and experimental q_e_ values for both dyes are given in Table 5. From this table, it was seen that the linear regression correlation coefficient (r) values were found to be higher (≥0.989) for MG and (≥0.967) for CV, and it was observed that the experimental (q_e,exp_) values were not in accord with the calculated (q_e,cal_) values obtained from the linear plots for both dyes. Frequently, in most studied sorption systems, the Lagergren pseudo-first-order equation does not fit well over the entire sorption period and is generally applicable over the first 20–30 min of the sorption process [21].

#### 2.3.2. Pseudo-Second-Order Model

The pseudo-second-order equation was first proposed by Blanchard et al. [44]. The pseudo-second-order expression (Equation (4)) can be linearized to six different linear forms [44,45], and the most used linearized form for the pseudo-second-order equation is given by the following equation.

(4) $$q_{t}=\frac{k_{2}q_{e}^{2}t}{1+k_{2}q_{e}t}$$

where k_2_ (g mg^−1^ min^−1^) is the rate constant for the pseudo-second order biosorption, and q_e_ (mg g^−1^) and q_t_ (mg g^−1^) are the amount of MG biosorbed at equilibrium and at any time t, respectively.

This model has been frequently employed to analyze biosorption data obtained from various experiments using different sorbates and biosorbents. The pseudo-second-order equation is based on the sorption capacity of the solid phase:

(5) $$\frac{t}{q_{t}}=\frac{1}{k_{2}q_{e}^{2}}+\frac{1}{q_{e}}t$$

The initial biosorption rate h (mg g^−1^ min^−1^) is given by the following equation:

(6) $$h=k_{2}q_{e}^{2}$$

**Figure 8:**
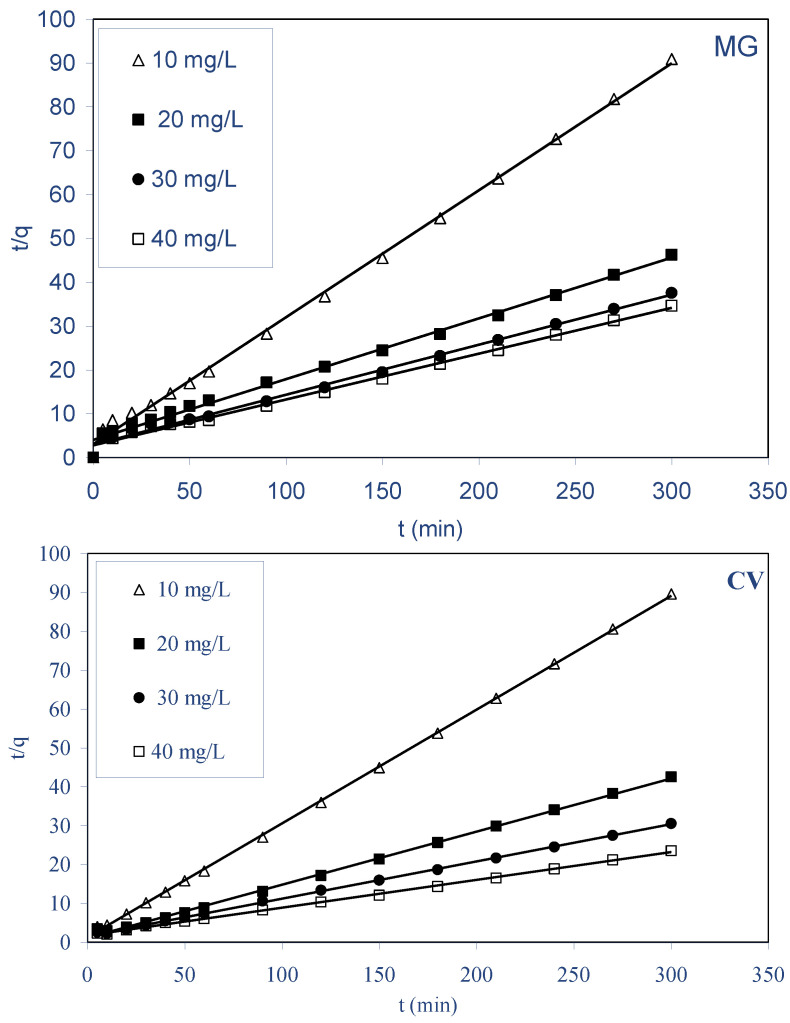
Pseudo-second-order kinetics for the biosorption of both dyes on ALP.

When this model is applicable, the plot of t/q_t_ against t should give a linear relationship, from which k_2_ and q_e_ could be determined from the intercept and slope of the plot, respectively. Figure 8 shows the pseudo-second-order plots for MG and CV biosorption by ALP at 298 K. The pseudo-second-order rate constants k_2_, the calculated biosorbed amount at equilibrium, q_e,cal_ values, the initial biosorption rate values h, and the corresponding correlation coefficients values (r) are collected in Table 5. For both dyes, the values of q_e,cal_ agree with the experimental biosorbed amount at equilibrium, q_e,exp_ values, and also, the correlation coefficients (r ≥ 0.994) at all the studied concentrations are very good. Then, the pseudo-second-order model predicts the biosorption behavior over the whole-time biosorption and the studied concentrations.

#### 2.3.3. Elovich Model

The biosorption of different contaminants from aqueous solutions on a variety of materials has been described using the Elovich equation [14,23,46]. The differential form of this equation is often expressed as:

(7) $$\frac{{\mathrm{dq}}_{t}}{\mathrm{dt}}=\propto e^{-{\beta q}_{t}}$$

where q_t_ is the sorption capacity at time t (mg g^−1^) and α is the initial sorption rate (mg g^−1^ min^−1^) because dq_t_/dt approaches α when q_t_ approaches zero, and β is the desorption constant (g mg^−1^). Given that q_t_ = 0 at t = 0, the integrated form of Equation (7) becomes:

(8) $$q_{t}=\frac{1}{\beta }\ln\left(t+t_{0}\right)-\frac{1}{\beta }\ln\left(t_{0}\right)$$

where t_0_ = 1/αβ. If t is much larger than t_0_, Equation (8) can be simplified to:

(9) $$q_{t}=\frac{1}{\beta }\ln\left(\alpha \beta \right)-\frac{1}{\beta }\ln\left(t\right)$$

Therefore, the Elovich kinetic constants could be deduced from the slopes and the intercepts of the linear plots of q_t_ against ln(t) (figure not shown), and the values of Elovich parameters were regrouped in Table 5. In the case of using the Elovich equation, the correlation coefficients are lower than those of the pseudo-second order equation and varying from 0.959 to 0.988 for MG and from 0.955 to 0.994 for CV. These results (Table 5) revealed that the Elovich equation is unsuitable to describe the experimental data for the biosorption of both dyes for higher initial concentrations but improves at lower dyes concentrations.

Consequently, the pseudo-second-order model can be used to predict the biosorption kinetics data for both dyes by ALP since the obtained correlation coefficients were higher than 0.994 and the values of q_e,cal_ agree with the experimental biosorbed amount at equilibrium, q_e,exp_ values for the entire period, and for all initial concentrations, while the Elovich model has the poorest fitting at high concentrations but improves at lower dyes concentrations (10 and 20 mg L^−1^) for both dyes on ALP.

### 2.4. Mechanism of Dyes Biosorption

Biosorption kinetics is habitually controlled by different mechanisms of which the most general were the diffusion mechanisms, which can be explained by the intraparticle diffusion model derived from Fick’s second law of diffusion, proposed by Weber–Morris [14]. In order to study the mechanism of both dyes’ biosorption by ALP, an intraparticle diffusion-based mechanism was studied. It is an empirically found functional relationship, common to most sorption processes, where uptake varies almost proportionally with the square root of time (t^1/2^) rather than with the contact time t [47]. The intraparticle diffusion model assumes that: (i) the external resistance to mass transfer is only significant for a very short period at the beginning of diffusion, (ii) the direction of diffusion is radial, and (iii) the pore diffusivity is constant and does not change with time [48].

**Figure 9:**
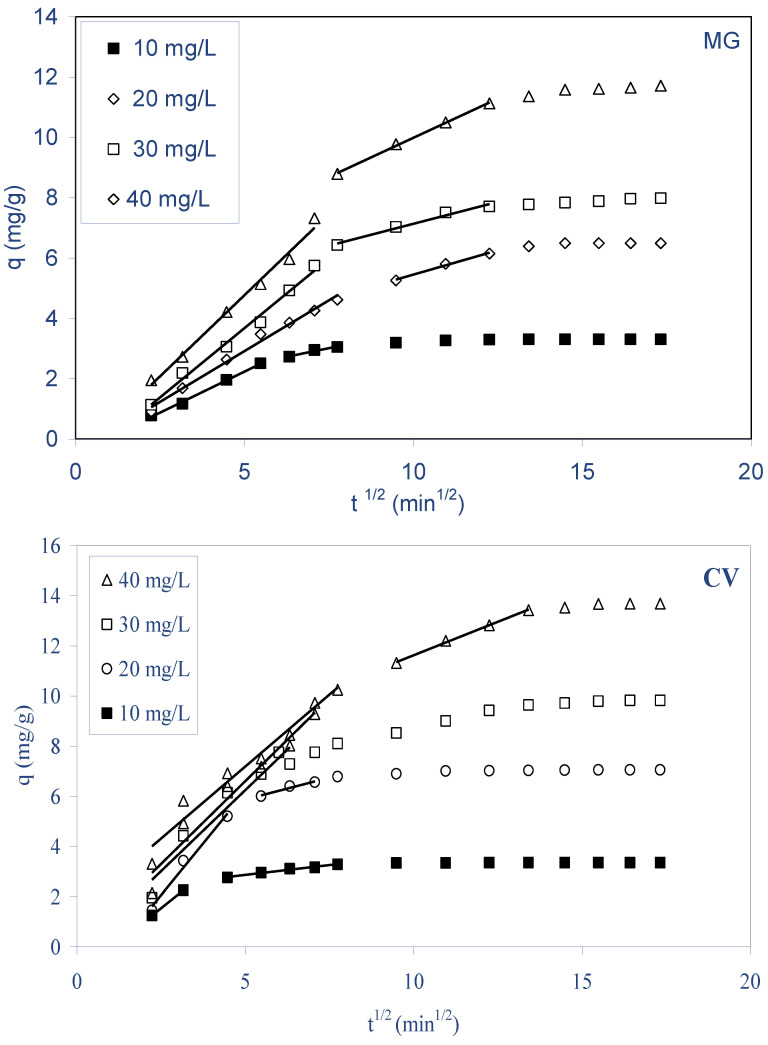
Intraparticle diffusion plots for the biosorption of both dyes on ALP (C_0_: 10–40 mg/L; biosorbent dosage: 2.5 g/L; stirring speed: 400 rpm; pH (MG): 4, pH (CV): 6.5 T: 298 K).

According to Equation (10), the intraparticle diffusion model is characterized by the relationship between specific sorption and t^1/2^:

q_t_ = k_d_ t^1/2^ + C_d_ (10)

where q_t_ is the sorbed amount at any time (mg g^−1^), k_d_ is the intraparticle diffusion rate constant (mg g^−1^ min^−1/2^), t^1/2^ is the square root of time (min^1/2^), and C_d_ is the intercept, which represents the resistance to mass transfer in the external liquid film (or the thickness of the boundary layer). Weber and Morris stated that if the regression of q_t_ versus t^1/2^ is linear and passes through the origin, then intraparticle diffusion is the sole rate-limiting step. Figure 9 shows the dependencies of q_t_ versus t^1/2^ for the biosorption of MG and CV by ALP at different initial dye concentrations at 298 K. From this figure, it was clear that the plots were multilinear, containing three consecutive linear steps with different slopes during the biosorption for both dyes by ALP, indicating the different periods in biosorption. The first linear period represents (1) the instantaneous biosorption or external surface biosorption; this can be attributed to the rapid use of the most readily available sorbing sites on the biosorbent surface. This is followed by (2) the second linear period, which is the progressive biosorption period where intraparticle diffusion is the rate limiting, and (3) the third period is the final equilibrium period where intraparticle diffusion starts to slow down due to the extremely low MG or CV dye concentrations in the solutions. It can also be observed from the results that the plots do not pass through the origin; this was indicative of some degree of boundary layer control, and this further showed that the intraparticle diffusion was not the only rate-limiting step, but other processes might implicate in control the rate of biosorption [49]. A similar observation was previously reported for the sorption of various cationic pollutants by different sorbents [22,47,50,51,52,53].

### 2.5. Biosorption Isotherms

The sorption isotherms revealed the specific relation between the concentration of the sorbate and its sorption degree onto sorbent surface at a constant temperature [25]. Various isotherm models have been used to describe the equilibrium data nature of biosorption. For this purpose, the biosorption equilibrium data for both dyes on ALP were modeled by Langmuir [54], Freundlich [23], Harkins–Jura [14,55], Flory–Huggins [14,23,56], Kiselev [54], and Elovich [23,54] equations. The applicability of the isotherm models to the biosorption study was judged by the correlation coefficient, r value, of each plot. The higher the r value, the better the fit [51].

The linear forms of these isotherm models (Equations (11)−(16)) were represented as follows:

(11) $$\begin{array}{cc} \mathrm{Langmuir} & \frac{C_{e}}{q_{e}}=\frac{1}{{\mathrm{bq}}_{m}}+\frac{1}{q_{m}}C_{e} \end{array}$$

(12) $$\begin{array}{cc} \mathrm{Freundlich} & \ln q_{e}={\mathrm{lnK}}_{F}+\frac{1}{n_{m}}{\mathrm{lnC}}_{e} \end{array}$$

(13) $$\begin{array}{cc} \mathrm{Harkins–Jura} & \frac{1}{q_{e}^{2}}=\frac{B}{A}+\frac{1}{A}{\mathrm{logC}}_{e} \end{array}$$

(14) $$\begin{array}{cc} \mathrm{Flory–Huggins} & \ln\frac{\theta }{C_{0}}={\mathrm{lnK}}_{\mathrm{FH}}+n_{\mathrm{FH}}\ln\left(1-\theta \right) \end{array}$$

(15) $$\begin{array}{cc} \mathrm{Elovich} & \ln\frac{q_{e}}{C_{e}}={\mathrm{lnK}}_{E}q_{m}-\frac{q_{e}}{q_{m}} \end{array}$$

(16) $$\begin{array}{cc} \mathrm{Kiselev} & K_{1}C_{e}=\frac{\theta }{\left(1-\theta \right)\left(1+K_{n}\theta \right)} \end{array}$$

where q_m_ is maximum biosorption capacity (mg g^−1^), b is Langmuir constant (L mg^−1^), K_F_ (mg^1−(1/n)^ L^1/n^ g^−1^) is Freundlich biosorbent capacity, n_F_ is a constant indicative of the intensity of the sorption (if n_F_ > 1, the biosorption is a favorable physical process), A and B were Harkins–Jura constants, θ is the degree of surface coverage, K_FH_ is the indication of Flory–Huggins equilibrium constant, n_FH_ is a constant model exponent, K_E_ is the Elovich equilibrium constant (L mg^−1^), q_m_ is the Elovich maximum adsorption capacity (mg g^−1^), K_1_ is the Kiselev equilibrium constant (L mg^−1^), and K_n_ is the constant of complex formation between sorbed molecules.

**Figure 10:**
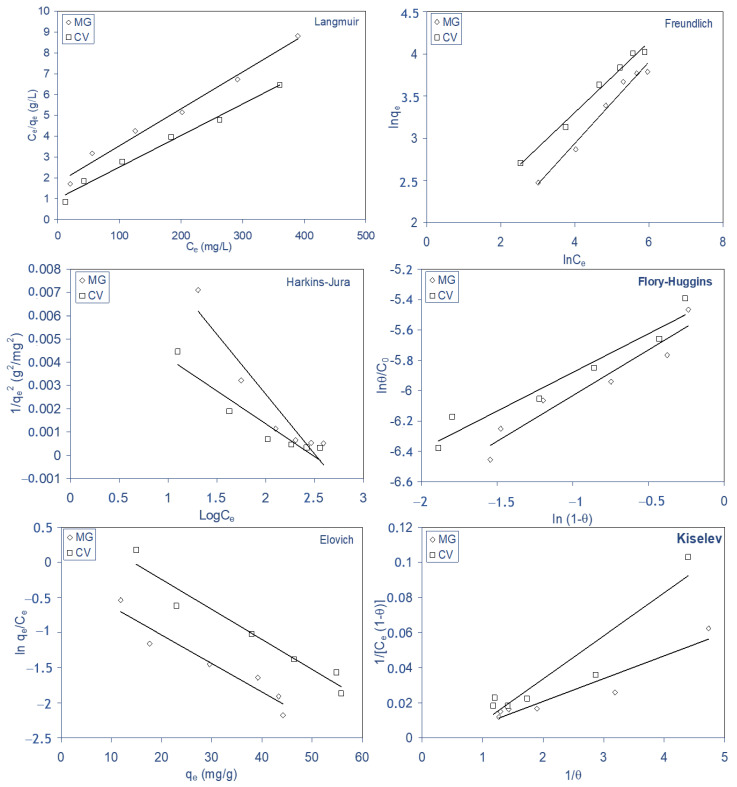
Linear plots of isotherm models for the biosorption of MG and CV on ALP: Langmuir, Frendlich, Harkins–Jura, Flory–Huggins, Elovich, and Kiselev.

The linear curves of these isotherm models from Equation (11) to Equation (16) to describe the experimental data at 298 K are represented in Figure 10, and the parameters of the models are consigned in Table 6. Based on the obtained correlation coefficient values (r), it was clear that Langmuir and Freundlich models provided a good fit (r ≥ 0.994) for the experimental equilibrium biosorption data for both dyes. The Langmuir model makes several assumptions, such as monolayer coverage and constant sorption energy, while the Freundlich equation deals with heterogeneous surface sorption [44]. The regression correlation coefficients are higher than 0.994 for both Freundlich and Langmuir models, signifying that both models closely fitted the experimental data, indicating that the biosorption of these dyes on ALP is monolayer biosorption and heterogeneous surface conditions exist under the used experimental conditions [44]. n_F_ > 1 indicates that the biosorption of these dyes on ALP is a favorable physical process. Similar isotherm results were already reported for the biosorption of MG on dead leaves of a plane tree [44]. The values of n_F_ were 2.1 and 2.7 for the biosorption of MG and CV, respectively, and the maximum biosorption capacity (q_m_) of Langmuir were 90.10 and 110.98 mg g^−1^, respectively, for MG and CV.

Table 7 lists the sorption capacity for MG and CV on ALP compared with that of several other low-cost sorbents reported in the literature. It should be mentioned that we cannot make a direct comparison for the maximum capacity of MG and CV by ALP compared to other sorbents (Table 7) because it does not have the same operating conditions, and also the characteristics of the sorbents are different. However, it was apparent that the ALP used in the present study has a relatively suitable biosorption capacity of 90.10 and 110.98 mg g^−1^, respectively, for MG and CV. Thus, it can be concluded that this agricultural waste can be used as an effective biosorbent material for the removal by biosorption of MG and CV from aqueous media.

In order to determine if the biosorption process is favorable or unfavorable, a dimensionless constant, commonly known as separation factor or equilibrium parameter (R_L_) defined by Webber and Chakkravorti [72], can be represented as:

(17) $$R_{L}=\frac{1}{1+{\mathrm{bC}}_{0}}$$

where b is the Langmuir constant and C_0_ is the initial concentration of the solute in solution.

**Figure 11:**
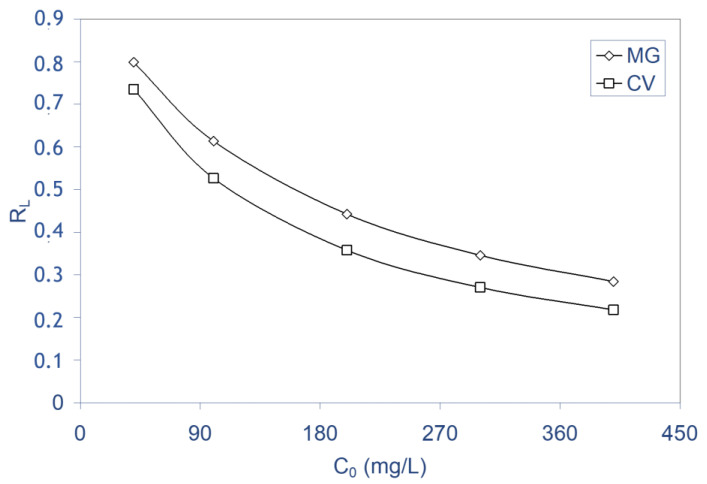
Separation factor (R_L_) for the biosorption of MG and CV on ALP at 298 K.

The parameter R_L_ indicated the shape of the isotherm as follows: R_L_ > 1 unfavorable, R_L_ = 1 linear, 0 < R_L_ < 1 favorable, R_L_ = 0 irreversible. The calculated R_L_ values vs. initial dye concentration at 298 K for MG and CV dyes were represented in Figure 11 and Table 6; from these, R_L_ values were obtained in the range of 0.79–0.28 for MG and of 0.73–0.21 for CV when the initial concentration is in the range of 40–400 mg L^−1^, respectively, indicating that the biosorption is a favorable process. Furthermore, it was seen that the biosorption of both dyes on ALP was found to be more favorable at higher concentrations. Such a finding is comparable to those made in earlier studies on the biosorption of pollutants by diverse biosorbents [5,23,44,47,68,73].

## 3. Materials and Methods

### 3.1. Preparation and Characterization of ALP as Biosorbent

The Alfa (*Stipa tenacissima* L.) leaves plant was collected from Djelfa (high plateaus of Algeria) with the stem with a length of 50–70 cm. The Alfa leaves plant was washed with water to remove the residues. The washed materials were then completely dried in an oven at 70 °C for 48 h and manually cut into small pieces, crushed, and sieved to obtain the powder of the Alfa leaf (ALP) with a desired size between 0.08–0.16 mm. Finally, the oven-dried materials were stored in airtight polyethylene bottles for sorption experiments. Principal characteristics of ALP used in the experiments were determined, and results are shown in Table 3.

The surface functional groups of ALP were determined by FTIR spectrometer (Shimadzu FTIR-8400S, Japan), the samples were prepared using the pressed potassium bromide (KBr) pellets containing 6% of ALP, and the obtained translucent pellets were analyzed in the range of 4000–400 cm^−1^. The titration of Boehm [74], which has been standardized [75], was used as a chemical technique to further identify the concentration of acidic and basic sites on the ALP surface. The point of zero charge (pH_pzc_) of the ALP was obtained by using the solid addition method [12]. Morphology analysis surface provides ocular insights into the surface of the sorbent at superior magnification. Scanning electron microscope (SEM) is used to study the surface morphology of the ALP sample with 1500× magnification before and after sorption. X-ray diffraction analysis was used to estimate the presence of crystalline phases present in the biosorbent. ALP crystal structure was characterized using an X-ray diffractometer equipped with a copper tube by scanning 2θ on powder samples.

### 3.2. Preparation of Dye Solutions

The dyes used were malachite green (MG) oxalate salt (C.I. 42,000; basic green 4, C_52_H_56_N_4_O_12_, FW 929) and crystal violet (CV) (C.I. 42,555, basic violet 3, C_22_H_30_N_3_Cl; FW 407.98). These dyes, purchased from Sigma-Aldrich, were chosen in this study as model molecules for organic pollutants in general and triphenylmethane basic dyes in particular. One hundred milligram per liter (1000 mg/L) stock solution was prepared by dissolving the required quantity of each dye in distilled water. Employed solutions of the chosen concentrations were obtained by consecutive dilutions. All reagents used in the present work were of analytical grade. MG and CV concentrations were determined by the measurement of the absorbance values after and before each test using Biochrom WPA Lightwave II UV-Vis. spectrophotometer at 618 nm and 586 nm, respectively.

### 3.3. Procedures of Biosorption

The biosorption experiments in batch mode were conducted in 150 mL conical flasks where 2.5 g/L of the biosorbent and 100 mL of each dye (10–40 mg/L) were added. The pH of all solutions in contact with the biosorbent was found to be around 4 and 6.5, respectively, for MG and CV, but for the effect of initial pH, the pH of each dye solution was adjusted by adding 0.1 N HCl or 0.1 N NaOH solutions using a pH meter (AD1030, ADWA instruments). The temperature effect was studied in the range of 298 to 328 K, and the biosorbent dosages were studied in the range of 2.5 to 5.5 g/L. The flasks were subsequently sealed and agitated magnetically at 400 rpm for 300 min to guarantee equilibrium was accomplished.

Samples were taken from each of the dyes’ solutions at fixed time intervals (0–300 min), and for each experiment, the solid phase was separated with centrifugation at 3000 rpm for 10 min, and the residual concentration present in the supernatant was determined by a UV–vis spectrophotometer.

Isotherm studies were carried out by contacting a fixed mass of ALP (0.2 g) with 80 mL of MG or CV solutions with initial concentrations of 40–400 mg L^−1^ and agitated magnetically at 400 rpm and different temperatures (298–328 K) for 24 h to ensure that equilibrium was attained for the higher concentrations. After equilibrium, samples of solutions were analyzed for the remaining dye concentration by using a UV-vis spectrophotometer.

## 4. Conclusions

The major conclusions of this study are drawn as follows:

- ALP can be used as an alternative biosorbent for cationic dyes MG and CV removal from aqueous solutions. The advantages of this biosorbent are high availability, low cost, and good biosorption capacity.
- The amounts of acid groups were higher than the basic groups, and this result determines the nature of the ALP surface that enhances the biosorption of the basic dyes from the aqueous phase. Additionally, the FTIR reveals the principal functional groups, which might be implicated in MG and CV biosorption such as OH of the phenol group of cellulose and lignin. The SEM of ALP displays that it is heterogeneous with the presence of spines and a rough, porous surface structure, which also improves the biosorption of dyes, and the X-ray diffraction technique indicates a characteristic shape of ALP that is amorphous in nature. The ALP presents a pH_pzc_ of 6.3.
- The biosorption process was influenced by a number of factors such as contact time and the initial dyes’ concentration, ALP biosorbent dose, initial pH of solution, temperature, and salt ionic strength concentration.
- For both dyes, the biosorption rate increased with increase in initial concentration, the biosorbent (ALP), temperature, initial pH of solution, and contact time, contrary to salt concentration, while stirring speed and total volume of the reaction mixture remained constant.
- The highest biosorption of both dyes was obtained at a pH greater than or equal to 6.
- For both dyes, the information obtained from biosorption isotherms at diverse temperatures (298–328 K) were employed to determine thermodynamic parameters such as ΔG˚, ΔH˚, and ΔS˚ of biosorption. The findings demonstrate that the biosorption is a physical process, spontaneous and endothermic in nature.
- The kinetics biosorption data for both dyes were found to obey to pseudo-second-order kinetics with a good correlation, and for the diffusion mechanism studies, the obtained results reveal that intraparticle diffusion is not the only rate-limiting step, but other processes may control the rate of biosorption for both dyes on ALP, and the equilibrium data were best described by the Langmuir and Freundlich isotherm model for both dyes. The maximum monolayer biosorption capacity was 90.10 and 110.98 mg g^−1^ for MG and CV, respectively, at 298 K.
- The dimensionless separation factor (R_L_) showed that ALP could be used for removal of cationic dyes (MG and CV) from aqueous media.

Subsequently, the results of this work will be valuable for using this agronomic waste as an economic biosorbent for basic dyes elimination in wastewater treatment procedures since it is accessible in larger amounts than other agricultural wastes.

## Figures and Tables

**Table 1:** Chemical composition of Algerian Alfa plant (fraction size: 0.25−0.5 mm) (adapted from [20]).

Component	Value
Ash	2.5
Extractives in:	
hot water	4.88
ethanol–toluene	6
ether	2.03
Lignin	24.3
Cellulose	44.1
Pentosans	26.8
Uronic acids	4.66

**Table 2:** Concentration of acidic and basic groups on ALP surface.

Concentration Groups	Carboxylic	Lactonic	Phenolic	Carbonylic and Quinonic	Acid	Basic	Total
Value (mequiv·g^−1^)	0.008	0.340	1.395	2.213	3.948	0.242	4.190

**Table 4:** Thermodynamic parameters for the biosorption of MG and CV on ALP.

Dye	T (K)	ΔG° (kJ/mol)	ΔH° (kJ/mol)	ΔS° (kJ/mol·K)
MG	298	5.52	1.99	25.3
	308		2.25	
	318		2.5	
	328		2.61	
CV	298	14.81	15.24	100.9
	308		16.25	
	318		17.32	
	328		18.25	

**Table 5:** Parameters of the kinetic models for the biosorption of MG and CV on ALP for different concentrations at 298 K.

Model	Initial MG Concentration (mg/L)				Initial CV Concentration (mg/L)			
Pseudo-first order	10	20	30	40	10	20	30	40
q_e_ (exp) (mg/g)	3.23	6.48	8.67	8.67	3.55	7.11	9.91	13.75
q_e_ (cal) (mg/g)	1.06	2.71	5.34	5.34	1.57	5.72	3.3	12.73
k_1_ (min^−1^)	0.02	0.021	0.025	0.025	0.04	0.045	0.021	0.032
r	0.981	0.997	0.988	0.988	0.978	0.972	0.968	0.967
Pseudo-second order	10	20	30	40	10	20	30	40
k_2_ (g/mg min)	0.062	0.027	0.004	0.038	0.076	0.019	0.006	0.009
q_e_ (cal) (mg/g)	3.45	6.2	8.77	11..56	3.6	7.34	9.45	13.93
h (mg/g min)	0.187	0.328	0.249	0.0403	0.888	1.696	0.696	0.908
r	0.999	0.998	0.996	0.994	0.999	0.999	0.998	0.997
Elovich	10	20	30	40	10	20	30	40
α (mg/g min)	0.778	0.635	0.613	0.801	1.815	0.583	0.947	0.271
β (g/mg)	0.342	0.445	0.501	0.628	0.364	0.531	0.562	1.312
r	0.982	0.988	0.959	0.969	0.994	0.982	0.955	0.97

**Table 6:** Comparison of maximum biosorption capacity (qm mg g^−1^) for CV and MG of some biosorbents in the literature.

	q_m_ (mg/g)	References
Crystal violet		
AC—from date palm leaflets	36.63	[1]
Modified Bambusa tulda	20.84	[9]
Termite feces	75.71	[57]
Banana peel	12.2	[58]
Coniferous pinus bark	32.78	[59]
Punica granatum shell	50.21	[60]
Sugarcane dust	3.42	[61]
Palm kernel fiber	78.9	[62]
Orange peel	14.3	[41]
Watermelon rind	104.76	[41]
Formosa papaya seed powder	85.99	[63]
Ricinus communis pericarp carbon	48	[64]
Jute fiber carbon	27.74	[65]
Coir pith	2.56	[66]
Alfa leaf powder (ALP)	110.98	This work
Malachite green		
Wheat bran	66.57	[2]
Rice bran	68.97	[2]
Potato peel	35.61	[12]
Dead leaves of plane tree	33.23	[44]
Luffa cylindrical	29.2	[46]
Coconut coir activated carbon	27.44	[67]
Rattan sawdust	62.71	[48]
Citrus limetta peel	8.73	[68]
Zea mays cob	16.72	[69]
Arundo donax root carbon	8.69	[70]
AC prepared waste apricot	116.27	[71]
Wood apple shell	34.56	[72]
Alfa leaf powder	90.1	This work

**Table 7:** Parameters of isotherm models for MG and CV biosorption on ALP at 298 K.

Element	Value	Element	Value
		MG	CV
Langmuir	q_m_ (mg/g)	90.1	110.98
	b × 10^3^ (L/mg)	6.3	8.99
	r	0.995	0.998
	R_L_ (40–400 mg/L)	0.79–0.28	0.73–0.21
Freundlich	K_F_	2.74	5.14
	n_F_	2.1	2.7
	r	0.994	0.998
Harkins–Jura	A	196.07	357.14
	B	2.531	2.721
	r	0.949	0.951
Flory–Huggins	K_F-H_ × 10^3^	4.65	4.38
	n_F-H_	0.51	0.602
	r	0.971	0.961
Kiselev	K_1_	0.013	0.0245
	K_n_	−0.04	−0.628
	r	0.932	0.943
Elovich	K_E_	0.032	0.078
	q_m_	24.63	23.47
	r	0.958	0.963

## Data Availability

Not applicable.
